# A novel fusion gene involving *PDGFRB* and *GCC2 *in a chronic eosinophilic leukemia patient harboring t(2;5)(q37;q31)

**DOI:** 10.1002/mgg3.591

**Published:** 2019-01-29

**Authors:** Noriyoshi Iriyama, Hiromichi Takahashi, Hiromu Naruse, Katsuhiro Miura, Yoshihito Uchino, Masaru Nakagawa, Kazuhide Iizuka, Takashi Hamada, Yoshihiro Hatta, Tomohiro Nakayama, Masami Takei

**Affiliations:** ^1^ Division of Hematology and Rheumatology, Department of Medicine Nihon University School of Medicine Tokyo Japan; ^2^ Division of Laboratory Medicine, Department of Pathology and Microbiology Nihon University School of Medicine Tokyo Japan; ^3^ Health Sciences Research Institute, Inc. Kanagawa Japan; ^4^ Division of Companion Diagnostics, Department of Pathology of Microbiology Nihon University School of Medicine

**Keywords:** chronic eosinophilic leukemia, *GCC2*, imatinib, *PDGFRB*

## Abstract

**Background:**

Platelet‐derived growth factor receptor beta (*PDGFRB*) rearrangement has been reported in a number of patients with chronic eosinophilic leukemia (CEL), B‐acute lymphoblastic leukemia, myeloproliferative neoplasms, and juvenile myelomonocytic leukemia. Here, we report a case of CEL carrying a novel fusion gene involving *PDGFRB* and GRIP and coiled‐coil domain containing 2 (*GCC2*).

**Patient and methods:**

A 54‐year‐old man presenting with a cough and dyspnea was diagnosed with acute eosinophilic pneumonia. Cytogenetic analysis of the bone marrow revealed the presence of t(2;5)(q37;q31). Fluorescence in situ hybridization analysis in the peripheral blood leukocytes revealed the presence of a split signal at *PDGFRB* gene. Imatinib treatment was effective, and disappearance of t(2;5)(q37;q31) in the bone marrow was confirmed after three months of imatinib therapy. Whole‐genome sequencing was performed in peripheral blood leukocytes collected before imatinib therapy.

**Results:**

A novel fusion gene between exon 22 of *GCC2* and exon 12 of *PDGFRB* was detected and the presence of *GCC2‐PDGFRB* was confirmed by PCR.

**Conclusion:**

This is the first case report demonstrating the *GCC2* gene as a partner of *PDGFRB* in the pathogenesis of CEL.

## 1 INTRODUCTION

Chronic eosinophilic leukemia (CEL) is a subtype of myeloproliferative neoplasms characterized by an increased number of eosinophils with blastoid cell proliferation or chromosomal abnormality involving platelet‐derived growth factor receptor alpha (*PDFGRA*; MIM #173490), platelet‐derived growth factor receptor beta (*PDGFRB*; MIM #173410), or fibroblast growth factor receptor1 (MIM #136350) (Apperley et al., 2002; Cools et al., 2003; Gotlib, 2017). CEL (including a subset of the hypereosinophilic syndrome [HES]) had been regarded to have a poor prognosis until the discovery of these fusion genes as potential targets of the tyrosine kinase inhibitor imatinib. This revolutionized the treatment of *BCR‐ABL1*‐positive chronic myeloid leukemia (Apperley et al., 2002; Gotlib, 2017; Hochhaus et al., 2017). CEL with *PDGFRA* or *PDGFRB* rearrangement is now recognized to be sensitive to imatinib, and the introduction of imatinib to the clinic has improved the treatment outcome in patients with this abnormality (Cheah et al., 2014; Gotlib, 2017; Jawhar et al., 2017).

Patients with *PDGFRA* rearrangement present with an increase in the number of eosinophils involving various organs such as lung, skin, guts, and nerves. *PDGFRB* rearrangement has been reported in a number of patients with CEL, B‐acute lymphoblastic leukemia, and myeloproliferative neoplasms with neutrophilia and/or monocytosis (Cheah et al., 2014; Gotlib, 2017; Helbig et al., 2010; Jawhar et al., 2017). The most common fusion partner for *PDGFRA* is *FIP1L1 *(MIM #607686*)*, which can be detected by fluorescence in situ hybridization analysis (FISH) (Apperley et al., 2002). Translocation with 12p13 (*ETV6*; MIM #600618) is reported to be present in 69% of patients with *PDGFRB*‐rearranged myeloid neoplasms (Cheah et al., 2014). However, other *PDGFRB* fusion partners including *WDR48, PDE4DIP, RAB5EP, PRKG2, SPTBN1, BIN2, TP53BP1, NUMA1, TSC1,* and *CEV14* have also been reported in myeloid neoplasms (Gong et al., 2016; Jawhar et al., 2017; Zhang et al., 2018; Zou et al., 2017). Although CEL with *PDGFRB* rearrangement is sensitive to treatment with imatinib, the clinical characteristics of these patients and the optimal treatment dose of imatinib are relatively unknown due to its rare incidence.

Here, we report a case of CEL with a novel fusion gene involving *PDGFRB* and GRIP and coiled‐coil domain containing 2 (*GCC2*; MIM #612711) and discuss the role of *GCC2* in the pathogenesis of CEL. This is the first report on the involvement of *GCC2* in hematologic neoplasms.

## 2 CASE REPORT

**Figure 1:**
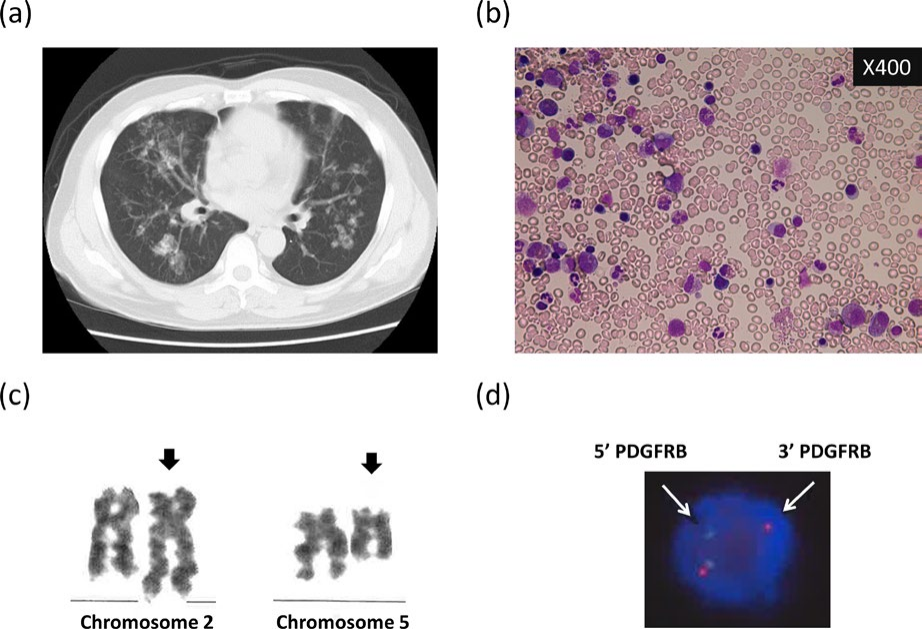
Computed tomography scanning showing the development of acute eosinophilic pneumonia (a). Morphology of bone marrow examination before imatinib therapy stained with May‐Giemsa (b). Cytogenetic analysis of bone marrow sample showing translocation between chromosomes 2q37 and 5q31 (c). This abnormality was observed in 16 of 20 metaphases. Fluorescence in situ hybridization of peripheral blood showing the presence of a split signal on platelet‐derived growth factor receptor beta (*PDGFRB)* gene (d). This was observed in 61% of nucleated cells

**Figure 2:**
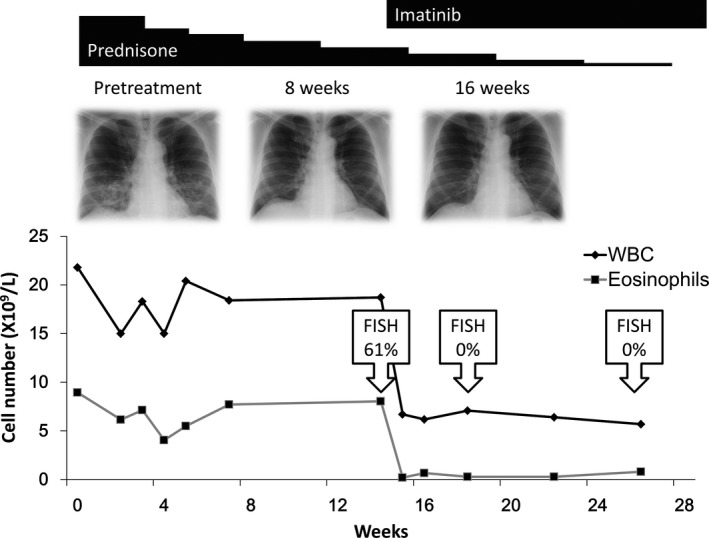
Clinical course of the patient after the incidence of acute eosinophilic leukemia. Low‐dose prednisone was initially administered, and it was effective for improving pneumonia but not in reducing the number of increased eosinophils. Imatinib administration rapidly reduced the number of eosinophils and the probability of cells harboring platelet‐derived growth factor receptor beta (*PDGFRB*) translocation in the peripheral blood. The probability of cells harboring *PDGFRB* translocation was evaluated by fluorescence in situ hybridization (FISH)

A 54‐year‐old man presenting with leukocytosis was referred to our hospital. Blood examination revealed eosinophilia—WBC 15.7 × 10^9^/L (neutrophils 28%, eosinophils 55%, basophils 1%, monocytes 3%, and lymphocytes 13%), Hb 13.0 g/dl, platelet count 339 × 10^9^/L, and LDH 232 U/L (normal range: 100–220). Liver and renal functions were normal. Since no clinical symptom or organ damage was identified, a regular monthly follow‐up was advised. After 4 months, he developed respiratory symptoms including cough and dyspnea. Chest X‐ray and computed tomography (CT) scanning revealed bilateral lung infiltrates (Figure 1a). Bronchoalveolar lavage fluid obtained by bronchoscopy revealed increased probability of eosinophils (20.5% eosinophils, 78.0% macrophages, 1.0% lymphocytes, and 0.5% neutrophils). He was diagnosed with acute eosinophilic pneumonia and was given prednisone at a dose of 0.5 mg kg^−1^ day^−1^. The clinical course of the patient is shown in Figure 2. Although treatment with prednisone improved the shadow of infiltrates on the X‐ray and the respiratory symptoms, it did not reduce the increased number of eosinophils in circulation. Therefore, bone marrow examination was carried out and it revealed normocellularity with elevated eosinophils (22.1% of nuclear cells) without blastoid cell proliferation (0%) (Figure 1b). Cytogenetic analysis of the bone marrow showed 46, XY, t(2;5)(q37;q31) [16/20]/46, XY [4/20] (Figure 1c). FISH analysis in the peripheral blood leukocytes revealed the presence of a split signal at *PDGFRB* (Figure 1d). In addition, WT1 mRNA was positively expressed (1,200 copies/μg RNA) in the peripheral blood.

After the detection of *PDGFRB* rearrangement, imatinib was given at a dose of 400 mg/day, since previous studies have reported a positive outcome from this dose in patients with hematologic neoplasms with *PDGFRB* rearrangement (Cheah et al., 2014; Jawhar et al., 2017). Imatinib treatment was effective, with rapid regression of eosinophils in the peripheral blood and the pneumonia shadow on lung X‐rays. The eosinophil number was back to normal after a week of imatinib therapy and the pneumonia shadow disappeared in 6 weeks. Translocation analysis by FISH also revealed the absence of *PDGFRB* rearrangement in the peripheral blood leukocytes after a month of imatinib treatment. The disappearance of t(2;5)(q37;q31) and a normal eosinophil count in the bone marrow were confirmed after 3 months. WT1 mRNA expression was negative (<50 copies/μgRNA) in the peripheral blood at that time. The dose of imatinib was reduced to 200 mg/day after 12 months of treatment. No recurrence was observed under imatinib therapy for over 4 years. No severe adverse effects were recorded—a grade 1 liver dysfunction, increased CPK level, anemia, renal dysfunction, and edema according to the Common Terminology Criteria for Adverse Events ver.4.0 were the only adverse events that developed during the observation period. This study was approved by the research ethics board of Nihon University School of Medicine in accordance with the Declaration of Helsinki (identifier 150–0) and written informed consent was obtained from the patient before sample analysis.

## 3 MATERIALS AND METHODS

### 3.1 Whole‐genome sequencing

Whole‐genome sequencing (WGS) was conducted on DNA sample extracted from whole leukocytes of peripheral blood obtained from the patient before imatinib therapy. Genomic DNA was extracted from the whole blood using Maxwell^®^ 16 LEV Blood DNA Kit (Promega, Fitchburg, WI), sheared into approximately 350 bp fragments, and used to make a library with TruSeq Nano DNA Sample Prep Kit (Illumina, San Diego, CA). Sequencing was performed on an Illumina HiSeq X platform in paired‐end 150 bp configuration.

### 3.2 Mapping and calling structural variations

Adapter sequences were removed by cutadapt (v1.2.1). After quality control, reads were mapped to the reference human genome (hg19) using BWA (ver.0.7.10). Mapping result was corrected using Picard (ver.1.73) for removing duplicates and GATK (ver.1.6‐13) for local alignment and quality score recalibration. Structural variation (SV) calls were performed using BreakDancer (ver.1.4.5). Annotations of SVs were based on RefSeq (UCSC Genome Browser, Feb 2017) and GENCODE (UCSC Genome Browser, ver. 19). SVs were further filtered according to the following criteria: (a) CTX (interchromosomal translocation), (b) the translocations between chromosomes 2 and 5, and (c) total number of supporting read pairs more than seven. The breakpoints of SVs were manually reviewed using Integrative Genomics Viewer (IGV).

### 3.3 PCR method

We designed oligonucleotide primers which were applicable to both amplification by PCR and direct sequencing by the dideoxy method. The forward primers were set 200 base‐pairs (bp) upstream from the breakpoints, and the reverse primers were set 200 bp downstream from the breakpoints. The following oligonucleotide primers were used for the detection of the fusion gene identified by WGS—forward 5′‐AAC AAC AAA CTA TGA TGT AGT TAG AG‐3′ and reverse 5′‐AGA GAA GGC AAG ACA CCA GCC CTA GGT‐3′.

The DNA used for WGS analysis was the same DNA that was used for direct sequencing. The concentration of genomic DNA was determined using a NanoDrop One^c^ Spectrophotometer (Thermo Fisher Scientific, Waltham, MA). The DNA was diluted to a final concentration of 100 ng/μL using nuclease‐free water. PCR was performed using a Veriti^TM ^200 thermal cycler (Thermo Fisher Scientific) with AmpliTaq Gold^® ^360 Master Mix (Thermo Fisher Scientific). Genomic DNA was used as the template with primers flanking the target gene. The PCR reaction conditions were 98°C for 3 m followed by 35 cycles of 98°C for 30 s, 60°C for 30 s, and 72°C for 30 s. Following PCR amplification, the amplification products were checked by agarose gel electrophoresis and purified using an ExoSAP‐IT purification kit for PCR products (Affymetrix/USB). The purification reaction conditions were 37°C for 15 m and 80°C for 15 m. Bidirectional sequencing was performed using forward and reverse primers. The reaction was carried out in a Veriti^TM ^200 thermal cycler using a BigDye^® ^Terminator v1.1 Cycle Sequencing Kit (Thermo Fisher Scientific). The reaction conditions were 98°C for 1 m followed by 25 cycles of 98°C for 10 s, 50°C for 5 s, and 60°C for 2 m. The sequencing reaction products were purified using a BigDye XTerminator^TM^ Purification Kit (Thermo Fisher Scientific). Capillary electrophoresis was performed using an Applied Biosystems^TM^ 3,130 DNA Analyzer (Thermo Fisher Scientific) and the obtained nucleotide sequence data were compared against sequence data deposited in databases, such as GenBank at NCBI.

## 4 RESULTS

### 4.1 Whole‐genome analysis

**Figure 3:**
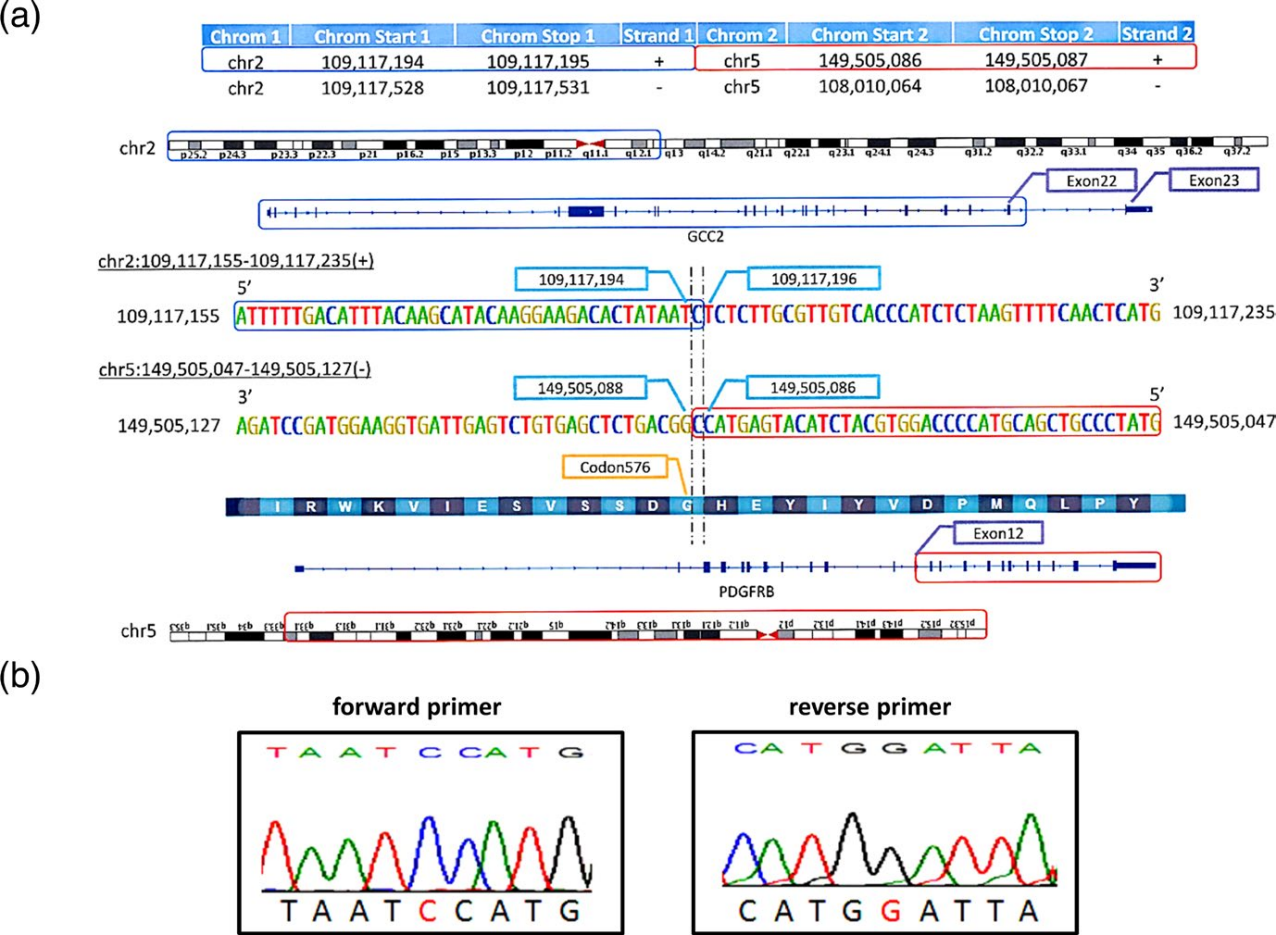
Whole‐genome sequence identifying the breakpoint forming platelet‐derived growth factor receptor beta (*PDGFRB*) and GRIP and coiled‐coil domain containing 2 (*GCC2*) fusion gene (a). A novel fusion gene between exon 22 of *GCC2* and exon 12 of *PDGFRB* was detected. Direct sequencing analysis confirming the presence of *GCC2‐PDGFRB* fusion gene (b)

First, we detected the variant for each case by WGS analysis using a next‐generation sequencer in whole leukocytes obtained from the patient before imatinib therapy. The results from the WGS analysis showed the definitive nucleotide sequences including the breakpoints constituting the fusion gene—46, XY, t(2;5)(q37;q31)/46, XY. As shown in Figure 3a, we were able to detect a novel fusion gene between exon 22 of *GCC2* and exon 12 of *PDGFRB*.

### 4.2 PCR analysis

Next, we confirmed the nucleotide sequence for these breakpoint sites by direct sequencing analysis. The result of the direct sequencing analysis was in agreement with the data from the WGS analysis. As shown in Figure 3b, the presence of *GCC2‐PDGFRB* fusion gene was confirmed by direct sequencing analysis.

## 5 DISCUSSION

In this study, we were able to detect a novel fusion gene comprising of *GCC2* (also called *GCC185*) and *PDGFRB*, involved in the pathogenesis of CEL. Results of G‐banding, FISH, WGS, and PCR analyses revealed the fusion of *PDGFRB* and *GCC2* genes. In addition, blood examination revealed a positive expression for WT1 mRNA before imatinib therapy which turned negative after remission, suggesting that WT1 mRNA level is a possible marker that could distinguish CEL from HES. Interestingly, WT1 mRNA appeared to be unsuitable for detecting minimal residual disease, since it became negative at the achievement of complete cytogenetic remission (indicated by the absence of chromosomal abnormality). Furthermore, the efficacy of imatinib therapy was excellent and triggered the rapid regression of eosinophils and pneumonia shadow on X‐rays in this case. However, PCR detection of the fusion gene was not possible since the fusion gene was unknown. Imatinib therapy was well tolerated and the patient is still under the treatment.

CEL with *PDGFRA* or *PDGFRB* rearrangement is usually sensitive to imatinib monotherapy in the chronic phase, ensuring long‐lasting remission (Helbig et al., 2010; Jawhar et al., 2017). Although the characteristics of CEL with *PDGFRA* rearrangement and that with *PDGFRB* rearrangement are similar, some differences have been reported. Both CEL with *PDGFRA* rearrangement and that with *PDGFRB* rearrangement are predominant in adult males. Both are sensitive to imatinib, but maintenance therapy is required to retain remission of the disease (Cheah et al., 2014; Gotlib, 2017; Helbig et al., 2010; Jawhar et al., 2017). The optimal treatment doses for these diseases are considerably different. According to a study that tested kinase inhibitory profiles, the IC_50_ of imatinib was 3.2 nM for *PDGFRA‐FIP1L1*, 50 nM for *ETV6‐PDGFRB*, and 582 nM for *BCR‐ABL1*. This demonstrates the differences in sensitivity to imatinib between these conditions (Apperley et al., 2002; Chen et al., 2004). This suggests the requirement for the determination of the ideal daily dose of imatinib therapy for CEL. According to the report by Helbig et al. (2008), very low dose imatinib therapy (100 mg/week) for CEL with *PDGFRA‐FIP1L1* fusion gene successfully maintained remission during the observation period. Patients with myeloid neoplasms with *PDGFRB* rearrangement have been treated with imatinib at a dose of 100–400 mg/day (Jawhar et al., 2017). Based on previous findings, we considered 100–200 mg/day to be the optimal therapeutic dose for CEL with *PDGFRB* rearrangement. Therefore, the dose of imatinib was reduced from 400 to 200 mg/day after cytogenetic remission (negative for *PDGFRB* rearrangement in the peripheral blood by FISH). After dose reduction, the patient sustained cytogenetic remission, suggesting that a dose of less than 200 mg/day is optimal for maintenance.

We found that the *GCC2* gene was a fusion partner for *PDGFRB* in this patient. The protein translated from the *GCC2* gene forms a part of the peripheral membrane protein localized to the trans‐Golgi networks (Luke, 2003). Although it is natural to consider that the *GCC2* gene has a critical role in the constitutive activation of PDGFRB in this patient, the association of GCC2 has rarely been reported in human diseases. Indeed, *GCC2* has never been described as a cause of a hematologic malignancy. However, in three patients with lung cancer, the *GCC2* gene was shown to fuse with anaplastic lymphoma kinase (*ALK*; MIM #105590), which has a critical role in the pathogenesis of non‐small cell lung cancer, and targeting it with crizotinib was shown to be active against cancer (Jiang et al., 2018; Noh et al., 2017; Vendrell et al., 2017). The non‐small cell lung cancer harboring *GCC2‐ALK* fusion gene supports the hypothesis that the *GCC2‐PDGFRB* gene products could behave as an oncoprotein. The products translated from the fusion gene are considered to be activated constitutively, which result in the stimulation of downstream pathways for eosinophil differentiation.

The mechanism underlying the PDGFRB‐mediated accumulation of eosinophils is not well understood. Some evidence has shown the activation of the downstream pathways by transfecting *PDGFRB* fusion gene into cells. Ishibashi et al. (2016) showed that the transfection of *ATF7IP‐PDGFRB* gene to Ba/F3 conferred IL3‐independent cell growth accompanied by the activation of MAP kinase and AKT. In addition, STAT1, STAT3, and STAT5 are also reported to be activated by *ETV6‐PDGFRB* transfection (Montano‐Almendras et al., 2012; Wilbanks et al., 2000). Furthermore, the requirement of nuclear factor‐kappaB (NF‐κB) for eosinophil differentiation and cell growth with *ETV6‐PDGFRB*‐transfectant has been previously reported (Montano‐Almendras et al., 2012). With respect to the significance of IL5‐dependent STAT5 activation for differentiation toward eosinophils in normal hematopoiesis, we speculate that multiple pathways other than STAT5, including STAT1, STAT3, MAPK, AKT, and NF‐κB, are critical participants for CEL development and progression. The investigation for the direct effect of imatinib on eosinophils in patients with CEL harboring *PDGFRA* and *PDGFRB* rearrangements is currently underway.

In conclusion, we here reported the first case of CEL demonstrating *GCC2* gene as a partner of *PDGFRB*. The functions of the fusion gene should be further investigated to clarify how it affects CEL pathogenesis.

## CONFLICTS OF INTEREST

N.I. and Y.H. received honoraria and speaker fees from Novartis Pharma K.K. The remaining coauthors declare no competing financial interests.
